# Organization Features of the Mitochondrial Genome of Sunflower (*Helianthus annuus* L.) with ANN2-Type Male-Sterile Cytoplasm

**DOI:** 10.3390/plants8110439

**Published:** 2019-10-23

**Authors:** Maksim S. Makarenko, Alexander V. Usatov, Tatiana V. Tatarinova, Kirill V. Azarin, Maria D. Logacheva, Vera A. Gavrilova, Igor V. Kornienko, Renate Horn

**Affiliations:** 1 Department of Genetics, Southern Federal University, Rostov-on-Don 344006, Russia; 2 The Institute for Information Transmission Problems, Moscow 127051, Russia; 3 Department of Biology, University of La Verne, La Verne, CA 91750, USA; 4 Vavilov Institute of General Genetics, Moscow 119333, Russia; 5 School of Fundamental Biology and Biotechnology, Siberian Federal University, Krasnoyarsk 660041, Russia; 6 Skolkovo Institute of Science and Technology, Moscow 121205, Russia; 7 The N.I. Vavilov All-Russian Institute of Plant Genetic Resources, Saint Petersburg 190121, Russia; 8 Southern Scientific Center of the Russian Academy of Sciences, Rostov-on-Don 344006, Russia; 9 Institute of Biological Sciences, Plant Genetics, University of Rostock, 18059 Rostock, Germany

**Keywords:** sunflower, cytoplasmic male sterility (CMS), mitochondrial genome, reorganizations, next generation sequencing (NGS)

## Abstract

This study provides insights into the flexibility of the mitochondrial genome in sunflower (*Helianthus annuus* L.) as well as into the causes of ANN2-type cytoplasmic male sterility (CMS). *De novo* assembly of the mitochondrial genome of male-sterile HA89(ANN2) sunflower line was performed using high-throughput sequencing technologies. Analysis of CMS ANN2 mitochondrial DNA sequence revealed the following reorganization events: twelve rearrangements, seven insertions, and nine deletions. Comparisons of coding sequences from the male-sterile line with the male-fertile line identified a deletion of *orf777* and seven new transcriptionally active open reading frames (ORFs): *orf324*, *orf327*, *orf345*, *orf558*, *orf891*, *orf933*, *orf1197*. Three of these ORFs represent chimeric genes involving *atp6* (*orf1197*), *cox2* (*orf558*), and *nad6* (*orf891*). In addition, *orf558*, *orf891*, *orf1197*, as well as *orf933*, encode proteins containing membrane domain(s), making them the most likely candidate genes for CMS development in ANN2. Although the investigated CMS phenotype may be caused by simultaneous action of several candidate genes, we assume that *orf1197* plays a major role in developing male sterility in ANN2. Comparative analysis of mitogenome organization in sunflower lines representing different CMS sources also allowed identification of reorganization hot spots in the mitochondrial genome of sunflower.

## 1. Introduction

Low substitution rate in genes along with considerable variability in size and structure are distinct features of plant mitochondrial genomes (mitogenome) [1,2]. Reorganization events in mitochondrial DNA (mtDNA) are primarily caused by disruption of a fragile equilibrium of intramolecular recombinations, maintained by nuclear-mitochondrial genetic interactions [3,4]. Runaway recombination of mtDNA can lead to changes in gene content and expression patterns of mitochondria [5,6]. The mitogenomes of flowering plants carry genes for rRNAs, tRNA, subunits of the respiratory chain complexes, as well as genes for the ribosomal proteins (*rps* and *rpl*). Maturase-related protein gene (*matR*) and genes responsible for the biogenesis of cytochrome *c* (*ccmB*, *ccmC*, *ccmFC,* and *ccmFN*) are also part of the plant mitochondrial gene set [7]. Alterations in transcription activity of the mitochondrial genes can have profound effects on the functionality of mitochondria and, thus, on different plant traits. Among the phenotypic traits caused by mitochondrial impairments, special attention is devoted to cytoplasmic male sterility (CMS) [8]. CMS is an inability to produce or shed functional pollen, which has been described in more than 140 higher plant species [9]. As a result of mtDNA rearrangements, new open reading frames are created, leading to male sterility [10]. In turn, dominant nuclear-encoded restorer-of-fertility genes (*Rf* genes) can restore normal development of pollen. Hence CMS/Rf systems are important both for studying pollen development in plants and for commercial applications [11,12]. The existence of the CMS phenotype in plants eliminates the need for laborious, manual emasculations for a directional crossing of plants, thus promoting its utilization in hybrid breeding [13]. 

Comparing mtDNA configurations in sunflower is especially interesting, as more than 70 CMS sources have been described within the Food and Agriculture Organization (F.A.O.) program for sunflower [14], even though modern sunflower hybrid breeding predominantly relies on a single male-sterile cytoplasm, the so-called CMS PET1 [15]. This CMS source originated from an interspecific cross of *H. petiolaris* with *H. annuus* [16]. The molecular characterization of the CMS mechanism helps to introduce new CMS sources into breeding programs. So far, the mitogenomes of only a few CMS sources have been sufficiently characterized to be used in sunflower hybrid production. The CMS phenotype can arise spontaneously in wild populations, while in breeding lines—after wide crosses, interspecific exchange of nuclear and/or cytoplasmic genomes, and mutagenesis [11]. It has been demonstrated that some CMS sources obtained from different inter- or intraspecific crosses showed the same mechanism of male sterility formation as the CMS PET1 type [17]. Even though these CMS sources had different origins, they have the same mitochondrial genome organization indicating that some configurations may be preferentially maintained in sunflowers [17].

Less is known about the spontaneously occurring CMS sources in *Helianthus annuus* L. [14]. ANN2 was derived from wild sunflower population N517 in Texas [18]. In this population, 40% of the plants were male-sterile [19]. However, sunflower cultivars with ANN2 CMS type, developed from the N517 population by maintaining with lines like HA89 or RHA265, showed 100% male-sterile progenies, which indicates a stable mitochondrial DNA configuration and absence of heteroplasmy. ANN2 and other spontaneously occurring CMS sources like ANN1, ANN3, and ANN4 maintained by RHA265 were hardly restored [20]. None of the tested maintainer and restorer lines of CMS PET1 were able to restore pollen production in CMS ANN1 and CMS ANN3. Only 12.5% and 15.8% of all investigated lines showed restorer capacity towards CMS ANN2 and CMS ANN4, respectively, indicating very different CMS mechanisms compared to CMS PET1. Three restorer lines, Rf ANN2-PI 413178, Rf ANN2-P21, and Rf ANN2-RMAX1, carry a restorer-of-fertility gene for ANN2 [21,22]. A suppressor gene S1 overpowering the restorer gene action has been recently described by Liu et al. [23], thus making the CMS-Rf interactions in the ANN2 source even more complicated.

Previous mtDNA investigations of some spontaneously occurring CMS sources were based on Southern blot hybridizations with mitochondrial genes [24]. Hybridizations of the CMS sources ANN1, ANN2, ANN3 with *atp6*, *atp9*, *cob*, *cox1*, *cox2*, *cox3*, *rrn18/rrn5/nad5*, *orfH522*, *orfH708*, or *orfH873* as a probe revealed unique banding patterns for 4 out of the 10 probes [24]. Besides, the analyses showed that CMS ANN4 and ANN5 are very similar to each other and form a distinct group from CMS ANN1, ANN2, and ANN3 [24]. It was also shown that ANN1/ANN2/ANN3 mtDNA-type significantly differs from both the male-fertile sunflower cytoplasm and the CMS PET1 source [24].

We describe the first assembly of the CMS source ANN2, which occurred spontaneously in *Helianthus annuus* L. The current study also provides insights into the flexibility of sunflower mitochondrial genome by comparing different isonuclear male-sterile lines HA89 (ANN2, MAX1, PET1, PET2) and the male-fertile line HA89, allowing identification of hot spots for rearrangements in the sunflower mitochondrial genome. For the CMS mechanism in ANN2, new open reading frames were identified, which were transcriptionally active. The ANN2 CMS source may be interesting not only for oilseed hybrid breeding, but also for horticultural purposes, as it is difficult to restore. It is a highly desirable trait in ornamental sunflowers since pollen production is usually not required nor looked-for, except if the pollen color enhances the contrast with the florets.

## 2. Results

### 2.1. Rearrangements in the Mitochondrial Genome of the Male-Sterile Line HA89(ANN2)

**Figure 1:**
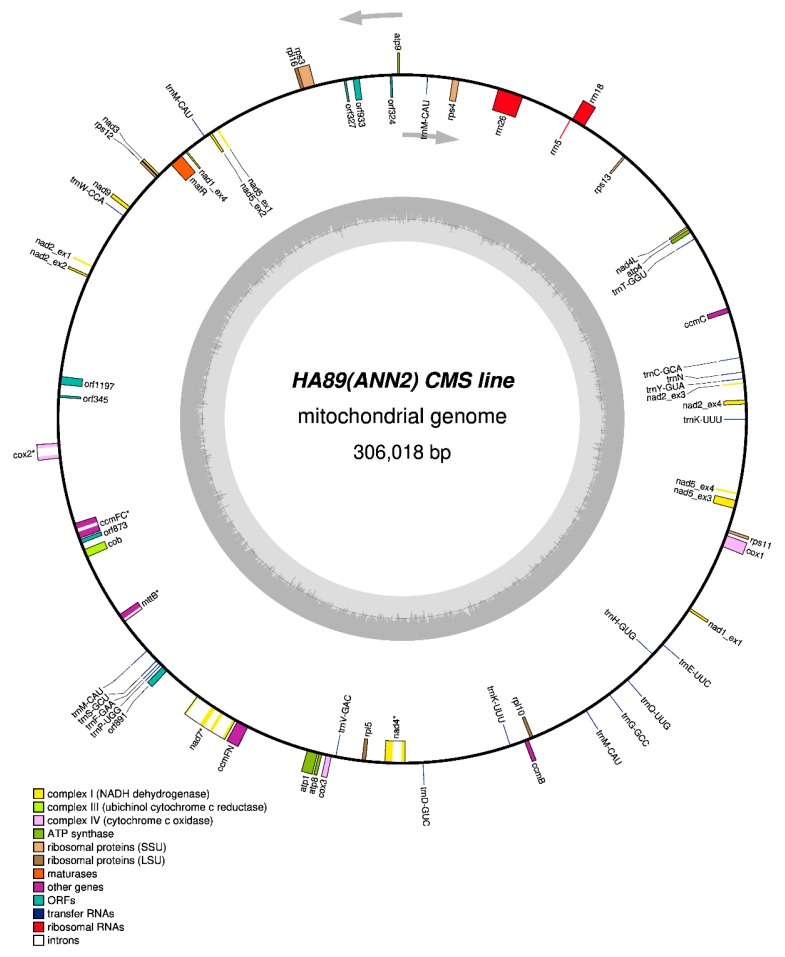
Mitochondrial genome map of HA89(ANN2) cytoplasmic male sterility (CMS) line of sunflower. Intron containing genes are marked by an asterisk (*) symbol. Trans-spliced genes are presented as the compilation of exons (ex).

We assembled the complete mitochondrial genome of the HA89 sunflower line with ANN2 cytoplasmic sterility type (NCBI accession MN175741.1). The master chromosome of HA89 (ANN2) consists of 306,018 bp (Figure 1), and it is 5071 bp longer than the mitogenome of the male-fertile isonuclear line HA89 (NCBI accession MN171345.1).

The HA89 (ANN2) mitochondrial genome has a wide range of rearrangements as compared to the male-fertile HA89 mitogenome. The summary of whole mitogenome alignment of male-sterile and male-fertile lines is presented in Table 1.

**Figure 2:**
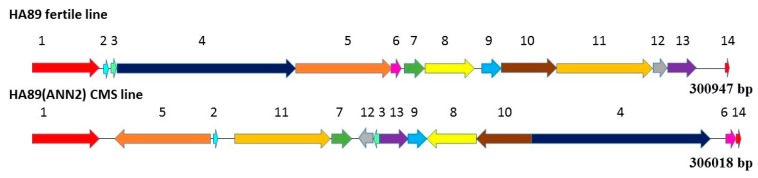
Schematic illustration of homologous regions between mitochondrial genomes of HA89 male-fertile and HA89(ANN2) CMS lines. 1—29,196 bp; 2—557 bp; 3—1245 bp; 4—77,441 bp; 5—41,702 bp; 6—4150 bp; 7—8584 bp; 8—21,433 bp 9—8158 bp; 10—24,687 bp; 11—41,505 bp; 12—6029 bp; 13—12,520 bp; 14—977 bp.

The mitochondrial genomes of male-sterile ANN2 and male-fertile HA89 share 14 complementary regions, but their localizations and orientation differ. We showed the localization of complementary regions in a scheme with both genomes shown in linear forms in Figure 2. Since regions #1 and #14 in the case of the circular molecule represent the same region, we classified the other twelve regions (#2–#13) as rearrangements.

In most cases, rearrangements only resulted in a reversal of a gene’s direction or a change in gene order. However, the 8584 bp (#7) and 21,433 bp (#8) rearrangements influenced the coding sequence of *nad6*, and the 6029 bp rearrangement (#12) impaired *atp6*. The largest part of the *nad6* gene sequence (~88%) is in the rearrangement #8, while the 3′ terminal part of *nad6* lies in the rearrangement #7. As a result of the convergence of #8 and #10 rearrangements in mitochondrial DNA of HA89(ANN2), the new *nad6*-chimeric open reading frame—*orf891*—was created. Analyses of *orf891* transcription pattern gave ambiguous results. Transcripts were detected for both *nad6* (HA89) and *orf891* (HA89(ANN2)) when using primers derived from the 5′ identical sequence of their mRNA (Supplementary Table S1). Nevertheless, using the same forward primer, but different reverse primers (Supplementary Table S1), complementary to the 3′ sequence of *nad6* and *orf891*, transcription was detected only for *nad6* (the fertile line), but not for *orf891* (CMS line). It is important to note that almost all the rearrangements found in mtDNA of HA89(ANN2) are accompanied by other types of genome reorganizations—deletions and insertions.

### 2.2. Deletions and Insertions in the Mitochondrial Genome of the Male-Sterile Line HA89(ANN2)

In comparison to the male-fertile analog, we identified nine longer than 100 bp deletions in the mtDNA of HA89(ANN2), which are shown in Table 2. Most deletions did not affect the protein-coding sequences, except for two deletions of 316 bp and 1165 bp. The 1165 bp deletion resulted in the total elimination of *orf777*, while the 316 bp deletion affected the part of the *atp6* gene. Interestingly, in previous studies, we also discovered the removal of *orf777* from the mitochondrial genomes of two other CMS lines—HA89(PET2) [25] and HA89(MAX1) [26].

Seven longer than 100 bp insertions were detected in mtDNA of the HA89(ANN2) CMS line (Table 3). As a result of these insertions in the mitochondrial DNA of HA89(ANN2), five new open reading frames, namely *orf324*, *orf327*, *orf345*, *orf558*, and *orf933*, have appeared. All five ORFs are transcribed in the case of ANN2, contrary to the HA89 line.

**Figure 3:**
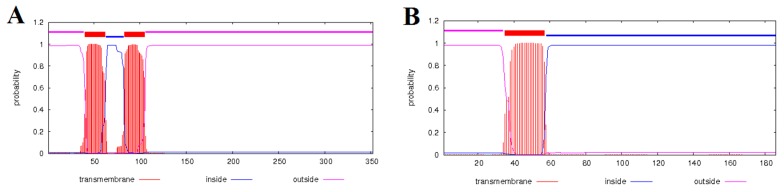
Comparison of transmembrane domains of proteins encoded by *cox2* (**A**) and *orf558* (**B**).

A search for transmembrane domains (TDs) revealed that the protein encoded by *orf558* contained a single TD. In the case of *orf933*, two TDs were detected. The *orf933* encoded protein did not show homology to other sunflower proteins in GeneBank, and had only limited similarity (40–60 amino acids) to hypothetical mitochondrial proteins with unknown functions in *Lactuca sativa* (accession PLY70338.1), *Salvia miltiorrhiza* (accession YP_008992338.1), *Beta vulgaris* (accession CBJ23356.1), etc. Forty-six amino acids of the N-terminus of the protein encoded by *orf558* matched the N-terminus of cytochrome c oxidase subunit 2 (*cox2* gene). Moreover, most of the amino acids that form the transmembrane domain in *orf558* protein are identical to those in COX2. However, the sunflower cytochrome c oxidase subunit 2 has two TD and the protein encoded by *orf558*—only a single one (Figure 3). So the *orf558* represents a chimeric *cox2* gene and could potentially play a role in the ANN2 CMS phenotype development. 

**Figure 4:**
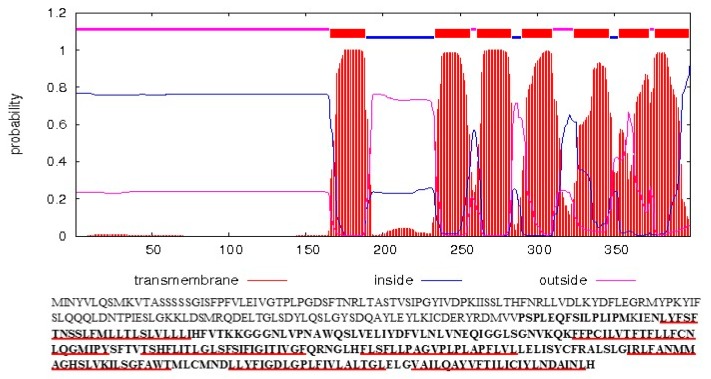
Comparison of *orf1197* and *atp6* encoded proteins and prediction of transmembrane helices. The amino acid sequence of the *orf1197* encoded protein is presented. Amino acids identical to ATP6 are shown in bold. Amino acids forming transmembrane domains are marked by red bars.

The most complex among the discovered ORFs in the HA89(ANN2) mitogenome was *orf1197*, which has appeared from three simultaneous reorganization events involving the 316 bp deletion, the 430 bp insertion, and the 6029 bp rearrangement. The *orf1197* represents a chimeric *atp6* gene, with transcription activity specific for the CMS line HA89(ANN2). In sunflower, the *atp6* gene typically encodes a protein consisting of 351 aa, whereas the predicted size of the translation product of the *orf1197* is 399 aa. Both proteins share 251 identical amino acids in the C-terminus. Thus, the protein encoded by the *orf1197* carries all seven TDs present in the C-terminus of the ATP synthase Fo subunit 6 from mitochondria of male-fertile sunflower (Figure 4).

## 3. Discussion

Recently, we had investigated complete mitochondrial DNA sequences for three CMS sources in sunflower, coming from interspecific crosses: PET1, PET2, MAX1 [25,26]. Comparison of the HA89(ANN2) mitogenome with mitochondrial genome assemblies of male-fertile lines [25,27] and the other HA89 male-sterile analogs provides insights into reorganizations of mtDNA associated with CMS phenotypes. The male-fertile lines (HA89, HA412) have only slight variations in the mtDNA sequence [25]. Whereas the mitogenomes of the CMS sources (HA89(ANN2), HA89(PET1), (HA89(PET2), HA89(MAX1)) showed significant differences as compared to their alloplasmic male-fertile analog. The complete mitochondrial genome of the male-fertile line HA89 adds up to 300,947 bp (NCBI accession MN171345.1), while HA89(PET1) has a size of 305,217 bp (NCBI accession MG735191.1), HA89(PET2) of 316,582 bp (NCBI accession MG770607.2), HA89(MAX1) of 295,586 bp (NCBI accession MH704580.1) and HA89(ANN2) of 306,018 bp (MN175741.1). The difference in genomes sizes is due to several deletions and insertions. For instance, in the mtDNA of all the investigated CMS sources, except HA89(PET1), a similar deletion in the *nad4L*-*orf777*-*atp8* region was observed. In the case of HA89(PET2), this is due to a 711 bp deletion, in HA89(MAX1) has a 978 bp deletion, and in HA89(ANN2) there is an 1195 bp deletion. All these deletions resulted in removal of *orf777* from the mtDNA in the CMS lines. Another region enriched by deletions is the area between *cob*-*ccmFC*, here three overlapping deletions were detected: a 451 bp deletion in HA89(PET1), one of 3780 bp in HA89(PET2) and another one of 4204 bp in HA89(ANN2).

**Figure 5:**
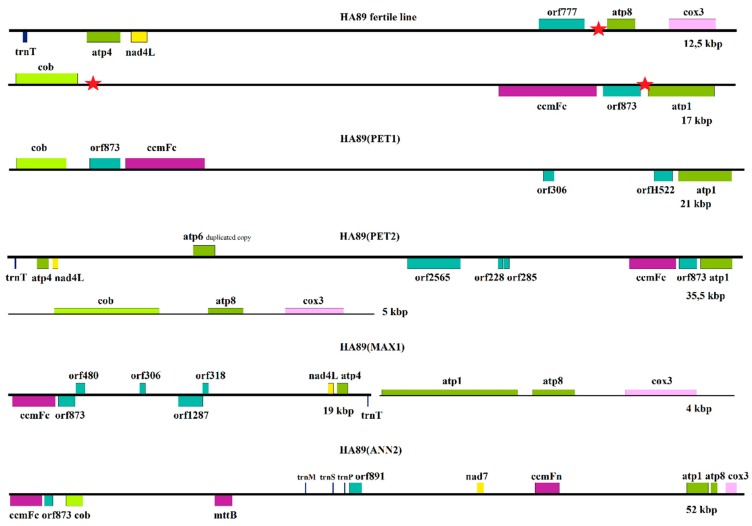
Reorganizations in CMS lines involving the 265 bp repeats (red stars) shown in the male-fertile mtDNA of HA89.

The coincidence between locations of these deletions is not accidental. There are three 265 bp repeats present in the sunflower mitochondrial genome, with the following positions in the mtDNA of the male-fertile HA89 line: 36537-36801 (adjacent to *atp8*), 190074-190338 (next to *cob*), and 202902-202638 (between *orf873*-*atp1*). These repeat regions are shown by red stars in Figure 5. Repeats represent common recombination points in mtDNA molecules [4,28]. The identification of small repeats involved in recombination is important because they influence the maintenance and evolution of mitochondrial genomes [28]. An imbalance in the nuclear-mitochondrion relationship that may occur in distant hybridizations impairs the recombination of mtDNA sub-genomic molecules, therefore, leading to reorganizations in the mitochondrial master chromosome. For instance, in HA89(PET1) the deletion, insertion, and inversion were mentioned in the *cob*-*atp1* region, directly between two repeats (Figure 5). In HA89(PET2), there were also several rearrangements in hot spots, resulting in the formation of a new gene cluster *cob*-*atp8*-*cox3*, as well as in the translocation of the *ccmFC*-*orf873*-*atp1* gene cluster into the *nad4L*-*orf777* region, combined with a deletion and huge insertion (Figure 5). In the mtDNA of both lines, HA89(MAX1) and HA89(ANN2), the specific *atp1*-*atp8*-*cox3* gene order was created, while in the MAX1 CMS source *ccmFC*-*orf873* translocated into the *nad4L*-*orf777* region (with deletion and huge insertion in this region). In the case of ANN2, the *ccmFC*-*orf873* region is located next to the *cob* gene (Figure 5). Thus, we have established that these three 265 bp repeats represent a reorganization hot spots in the sunflower mitochondrial genome.

Considering the insertions discovered in the HA89(ANN2) mitogenome, we also observed a similarity between the insertions of different CMS sources in sunflower. For instance, about 85% of the 1027 bp insertion sequence (ANN2) is complementary to the part of the 15,885 bp insertion (PET2). The 3757 bp insertion (ANN2) contains 1959 nucleotides identical to the 15,885 bp insertion (PET2) and 1215—to the 5272 bp insertion (MAX1). Also, 2343 bp of the 5338 bp insertion in ANN2 are similar to another region of sunflower mtDNA proximal to the *cob* gene (position 185,987–188,330 in the mtDNA of HA89 fertile line). So, this sequence is duplicated in the mitochondrial genome of the HA89(ANN2) CMS line. About 10% of 6452 bp insertion (ANN2) is complementary to both the 5050 bp (PET2) and the 5272 bp (MAX1) insertions. As well as 1158 bp of the 9044 bp insertion (ANN2) are identical to the 5050 bp and 15,885 bp insertions (PET2) and 5272 bp insertion (MAX1).

Although there are similarities in deletions, insertions, and rearrangements between mitochondrial genomes of the HA89(ANN2) and other CMS lines, the discovered ORFs were different. We summarized the data of all identified ORFs in the male-sterile cytoplasms in Table 4. The ORFs encoding proteins with similarity to other mitochondrial proteins and especially having transmembrane domains are of particular interest since the chimeric proteins with TD most often cause CMS phenotypes [10,29]. In the mtDNA of HA89(ANN2), we detected three new transcriptionally active ORFs, encoding proteins with TD—*orf558* (one TD), *orf933* (two TD), *orf1197* (seven TD). The *orf933* shows no homology to other sunflower genes, while *orf558* represents a chimeric *cox2* gene, and *orf1197* a chimeric *atp6* gene. It is difficult to estimate the exact contribution of *orf558*, *orf933*, *orf1197* to the development of the male-sterile phenotype in ANN2. However, the possibility of involvement of more than one open reading frame might be one explanation that ANN2 is so difficult to restore. On the other hand, the presence of a suppressor gene S1 discovered by Liu et al. [23] might be the reason for low rates of fertility restoration of the ANN2 CMS source. Previous studies indicate that the chimeric *atp6* genes or new ORFs that are co-transcribed with *atp6* most often cause CMS phenotypes in flowering plants [10,30,31,32,33]. Therefore, we suggest that *orf1197* is the major CMS candidate gene for ANN2 CMS source. Moreover, chimeric *atp6* genes were also identified in MAX1 [26] and CMS3/ANT1 [34] CMS types of sunflower. In CMS lines, chimeric *atp6* genes encode N-terminal extended proteins compared to the normal ATP synthase subunit 6 (351 aa): ANN2—399 aa, MAX1—429 aa, (AYV91168.1), CMS3/ANT1—437 aa (CAA57790.1). Moreover, 397 of 399 amino acids in the *orf1197* protein are identical to the chimeric *ATP6* of CMS3/ANT1 line, and therefore this protein represents a shorter version of this 437 aa-long protein. Such similarities support our hypothesis about the importance of *orf1197* in shaping the CMS phenotype in ANN2.

The *orf558* (as the chimeric *cox2* gene) might also cause cytoplasmic male sterility in ANN2. In other plants species, modified *cox2* sequences seem to be involved in the male sterility. For instance, the CMS specific *pcf* gene in petunia is composed from sequences of the 5′ portion of *atp9*, segments of *cox2*, and a large region of unknown origin—*urfS* [35]. In wild beets, the CMS-associated *orf129* shows homology to the 5′ flanking and the coding sequence of *cox2* [36]. In the mitogenome of the inap CMS source of *Brassica napus*, which was created by a somatic hybridization with *Isatis indigotica*, a novel *cox2-2* gene was detected, which represents recombination of the *cox2* of woad and *cox2-2* of rapeseed [37].

Another unique feature of HA89(ANN2) mitogenome is the formation of *orf891*. According to the ORFs predictions, a 3′ elongation of the *nad6* gene (*orf891*) may occur. However, the cDNA analyses did not agree with the genomic data. Perhaps due to *nad6* mRNA editing instead of a 3′ elongated transcript, the shorter one is formed. Heteromorphism in *nad6* transcript length was also observed in *Mimulus guttatus x M. nasutus* hybrids with the CMS phenotype [38]. Both male-fertile and male-sterile hybrids have a single copy of the *nad6* gene, and the divergence in mRNA length was only observed in male-sterile plants [38]. 

## 4. Materials and Methods

### 4.1. Plant Material

The CMS line HA89(ANN2) of sunflower was obtained from the genetic collection of the N. I. Vavilov All-Russian Institute of Plant Genetic Resources (Saint Petersburg, Russia). The original source of the ANN2 male-sterile cytoplasm was obtained by Serieys in 1984 [18]. All sunflower lines were grown in regularly irrigated pots in the growth chamber KBWF 720 (Binder, Tuttlingen, Germany) with the following growth conditions: temperature—26 °C, humidity—70%, photoperiod—10/14 h (dark/light). 

### 4.2. Mitochondrial DNA Extraction, NGS Library Preparation, and Sequencing

First, the organelle fraction from leaves of 14-days-old sunflower seedlings was isolated, as described by Makarenko et al. [39]. Such preparations significantly reduced the amount of nuclear DNA. Then DNA extraction was performed with PhytoSorb kit (Syntol, Moscow, Russia), according to the manufacturer’s protocol. Equal amounts of DNA from seven plants were mixed, and we used 1 ng of DNA pull for the NGS library preparation step. The library was made with Nextera XT DNA Library Prep Kit (Illumina, Mountain View, CA, USA), following the guidelines of Illumina. The quality of the library was evaluated using Bioanalyzer 2100 (Agilent, Santa Clara, CA, USA). The library was quantified at the Qubit fluorimeter (Invitrogen, Carlsbad, CA, USA) and by qPCR, then diluted up to the concentration of 8 pM. Sequencing was performed on two different Illumina sequencing platforms: HiSeq 2000 using TruSeq SBS Kit v3-HS 200-cycles and MiSeq using MiSeq Reagent Kit v2 500-cycles (Illumina, Mountain View, CA, USA). A total number of 3,063,836 reads (100-bp paired) and 3,305,268 reads (250-bp paired) were generated.

### 4.3. Mitochondrial Genome Assembly and Annotation

Quality control of reads was done using FastQC (https://www.bioinformatics.babraham.ac.uk/projects/fastqc/). Trimming of adapter-derived and low quality (Q-score below 25) reads was performed with Trimmomatic software [40]. For contig generation, we used SPAdes Genome Assembler v.3.10.1 [41]. The whole mitochondrial genome was manually assembled using scaffolds based on high coverage (depths > 70) contigs (length > 1000 kbp) and available bridge contigs (length = 0.3–1 kbp). The genome assembly was validated by remapping the initially obtained reads using Bowtie 2 v.2.3.3 [42]. All observed rearrangements were verified by PCR analysis. For variant calling, we used samtools/bcftools software [43] and manually revised polymorphic sites using the IGV tool [44].

The mitochondrial genome was annotated with MITOFY [45], BLAST tool [46], and ORFfinder (https://www.ncbi.nlm.nih.gov/orffinder). Using GeSeq [47], we provided comparisons of our annotation with the current reference annotations (NCBI accessions NC_023337.1, CM007908.1) of the sunflower mitochondrial genome. Graphical genome maps were generated using the OGDRAW tool v.1.3.1 [48]. The prediction of transmembrane domains was made with TMHMM Server v.2.0 (http://www.cbs.dtu.dk/services/TMHMM-2.0/). The scheme of the bioinformatic pipeline is presented in Supplementary Figure S1.

### 4.4. Expression Analyses

RNA was extracted from leaves of seven 28-days-old sunflower plants using a guanidinium thiocyanate-phenol-chloroform based method with the ExtractRNA reagent kit (Evrogen, Moscow, Russia). The quality and concentration of the RNA were evaluated with the Qubit fluorimeter (Invitrogen, Carlsbad, CA, USA) and the NanoDrop 2000 spectrophotometer (Thermo Fisher Scientific, Waltham, MA, USA). Total RNA (0.5 μg) was treated with DNAse I (Thermo Fisher Scientific, Waltham, MA, USA), and then cDNA was synthesized using the MMLV RT kit (Evrogen, Moscow, Russia) with random primers. As a negative control for each DNAse treated mRNA sample, the same reverse transcription protocol was performed, but without the MMLV enzyme. The quantitative PCR was performed with EvaGreen based RT-PCR kit (Syntol, Moscow, Russia) on Rotor-Gene 6000 (Corbett Research, Mortlake, NSW, Australia). A summary of all primer sequences is given in Supplementary Table S1. The primers annealing temperature (Tm) was 60 °C.

## 5. Conclusions

Assembly of CMS ANN2 mitochondrial genome (HA89(ANN2) line) revealed several rearrangements, insertions, and deletions, as well as seven new open reading frames: *orf324*, *orf327*, *orf345*, *orf558*, *orf891*, *orf933*, and *orf1197*. Transcripts were detected for all new open reading frames in CMS ANN2, but not in the fertile cytoplasm. Only *orf558*, *orf891*, *orf933*, and *orf1197* encoded proteins that contained membrane domains, making them the most likely CMS candidate genes for the ANN2 source. Notably, *orf1197* represents a chimeric *atp6* gene and presumably plays a major role in the CMS phenotype development of ANN2. However, CMS ANN2 may be caused by simultaneous action of several candidate genes. Hot spots for rearrangements (265 bp repeats) were identified, and we propose that they influence the maintenance and evolution of the mitochondrial genome in sunflower.

## Figures and Tables

**Table 1:** Alignment of the mitochondrial genomes of HA89 and HA89(ANN2) lines.

№ of Alignment Region	The Alignment Region Length, bp	Positions in mtDNA of Male-Fertile HA89	Positions in mtDNA of Male-Sterile HA89(ANN2)	Orientation	Similarity %	Localized Genes
1	29,196	1–29,196	1–29,204	Plus/Plus	99	*nad2_ex3,4*, *trnY*, *trnN*, *trnC*, *ccmC*, *trnT*, *atp4*, *nad4L*
2	557	33,772–34,328	78,343–78,899	Plus/Plus	99	-
3	1245	34,329–35,573	148,163–149,411	Plus/Minus	98	-
4	77,441	36,739–114,179	217,575–295,553	Plus/Plus	95	*atp8*, *cox3*, *trnV*, *rpl5*, *nad4*, *trnD*, *trnK*, *ccmB*, *rpl10*, *trnM*, *trnG*, *trnQ*, *trnH*, *trnE*, *nad1_ex1*, *cox1*, *nad5_ex3,4*, *rps11*
5	41,702	114,180–155,882	35,657–77,315	Plus/Minus	99	*atp9*, *trnM*, *rps4*, *rrn26*, *rrn5*, *rrn18*, *rps13*, *nad1_ex2,3*
6	4150	155,883–160,032	300,892–305,041	Plus/Plus	99	-
7	8584	160,320–168,903	129,358–137,946	Plus/Plus	99	*nad2_ex1,2*, *nad6* *
8	21,433	168,906–190,275	171,388–192,871	Plus/Minus	98	*nad6* *, *trnP*, *trnF*, *trnS*, *trnM*, *mttB*, *cob*
9	8158	194,543–202,700	163,232–171,387	Plus/Plus	99	*ccmFC*, *orf873*
10	24,687	202,701–227,387	192,915–217,574	Plus/Minus	99	*atp1*, *ccmFN*, *nad7*
11	41,505	227,396–268,900	87,945–129,446	Plus/Plus	99	*nad1_ex4*, *rps3*, *rpl16*, *trnM*, *matR*, *nad3*, *rps12*, *nad9*, *trnW*, *nad5_ex1,2*
12	6029	269,217–275,245	141,704–147,732	Plus/Minus	99	*atp6* *
13	12,520	275,536–288,055	150,723–163,231	Plus/Plus	99	*cox2*
14	977	299,971–300,947	305,042–306,018	Plus/Plus	99	*trnK*

* genes, which had impaired sequences as a result of rearrangements.

**Table 2:** Deletions (>100 bp) localized in the mitochondrial genome of HA89(ANN2) CMS line.

Deletion Length, bp	Positions in mtDNA of the Male-Fertile Line HA89	Deletion Localization according to the Male-Fertile Line HA89 Genetic Map	Deleted Genes
287	160,032–160,319	*rps13-nad6*	-
290	275,246–275,535	*atp6-cox2*	-
299	56,701–56,999	*nad4-ccmB*	-
316	268,901–269,216	*nad9-atp6*	*atp6* (partial)
583	70,338–70,920	*rpl10-nad1*	-
1165	35,574–36,738	*nad4L-orf777-atp8*	*orf777*
4204	190,339–194,542	*cob-ccmFC*	-
4575	29,197–33,771	*nad4L-orf777*	-
11,901	288,070–299,970	*cox2-nad2*	-

**Table 3:** Localization of insertions (>100 bp) in the mitochondrial genome of HA89(ANN2) CMS line.

Insertion in bp	Positions in mtDNA of HA89(ANN2)	New ORFs Based on Insertion	Homology to
430	147,733–148,162	*orf1197* *	*atp6*
1027	77,316–78,342	*orf324*	*orf285* (CMS PET2)
1310	149,412–150,722	*orf345*	
3757	137,947–141,703		
5338	295,554–300,891	*orf558*	*cox2*
6452	29,205–35,656		
9045	78,900–87,944	*orf327*, *orf933*	

* appeared as the result of several simultaneous reorganizations of mtDNA structure.

**Table 4:** Summary of transcriptionally active open reading frames in the mitochondrial genome of isonuclear fertile and CMS lines.

Fertile mtDNA	CMS ANN2	CMS MAX1	CMS PET2	CMS PET1
-	-	-	***orf228***	-
-	-	-	***orf285***	-
-	-	*orf306*	-	*orf306*
-	*orf324*	-	-	*-*
-	*orf327*	-	-	-
-	*orf345*	-	-	-
-	-	*orf480*	-	-
-	-	-	-	***orfH522***
-	***orf558***	-	-	-
-	-	*orf645*	*orf645*	-
*orf777*	-	-	-	*orf777*
-	***orf891***	-	-	-
-	***orf933***	-	-	-
-	***orf1197***	-	-	-
-	-	***orf1287***	-	-
-	-	-	*orf2565*	-

**In bold**: ORFs encoding proteins with transmembrane domains.

## Supplementary Materials

The following are available online at https://www.mdpi.com/2223-7747/8/11/439/s1, Suppl Table S1. The primers sets used for transcription activity analysis, Suppl Table S2. The protein coding genes annotated in HA89(ANN2) mitochondrial genome, Suppl Figure S1. Bioinformatic pipeline for mitochondrial genome analysis. Trimmed reads are available at https://doi.org/10.6084/m9.figshare.8945084.v1.
