# Supplementary material for: Organization Features of the Mitochondrial Genome of Sunflower (Helianthus annuus L.) with ANN2-Type Male-Sterile Cytoplasm
Source: Plants (Basel). 2019 Oct 23;8(11):439. doi: 10.3390/plants8110439 (PMC6918226; doi:10.3390/plants8110439)
Supplement: Supplementary file 1 [file plants-08-00439-s001.pdf]

Suppl Figure 1. Bioinformatic pipeline for mitochondrial genome analysis. Trimmed reads are available at https://doi.org/10.6084/m9.figshare.8945084.v1.

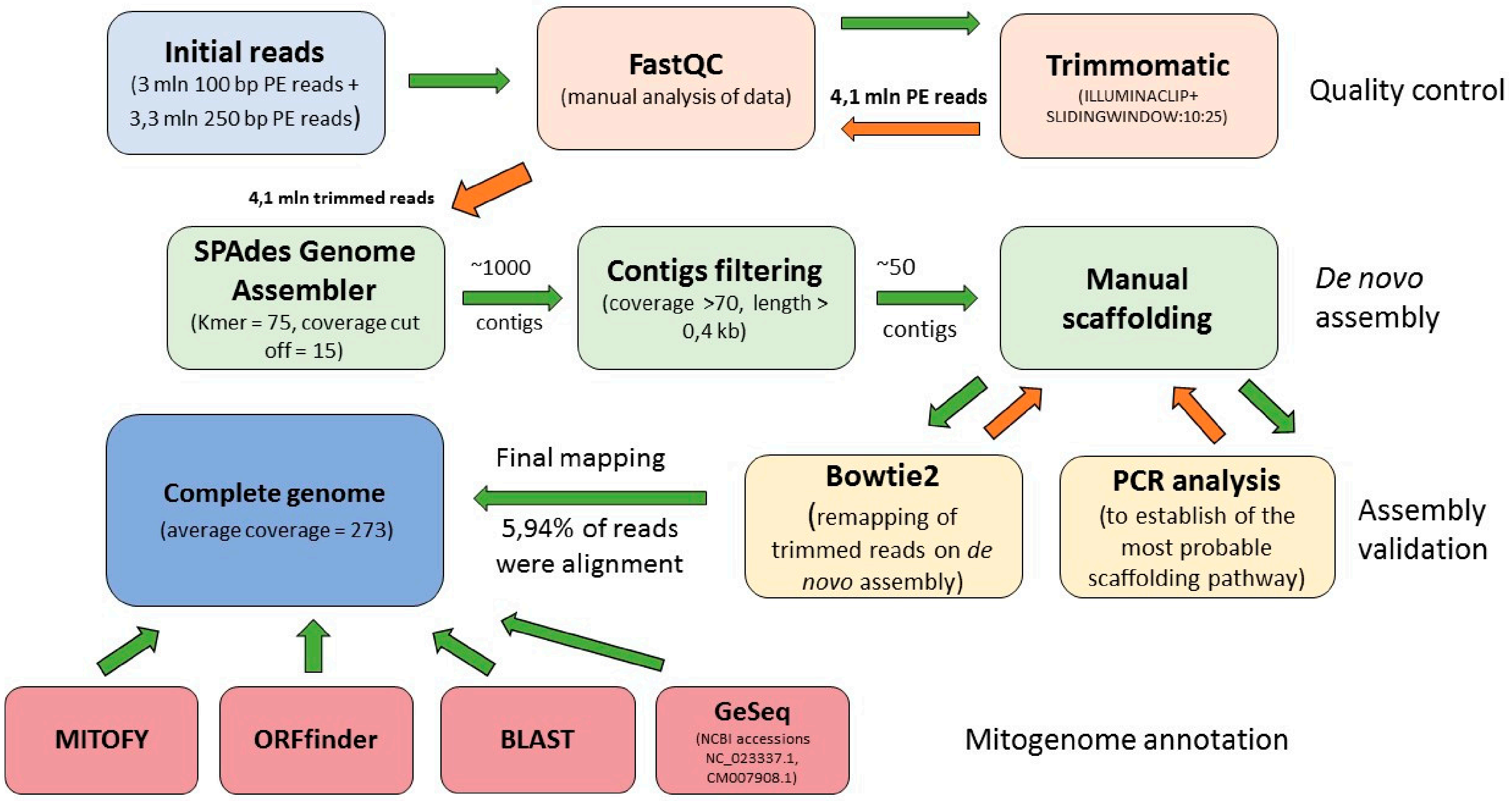

Suppl Table 1. The primers sets used for transcription activity analysis.

Gene	Forward Primer Sequence (5′-3′)	Reverse Primer Sequence (5′-3′)	The Amplicon Size (bp) in HA89 Male-Fertile Line	The Amplicon Size (bp) in HA89(ANN2) Male-Sterile Line
orf324	GAACCACCTTTCGCTAACCT	CATCGACTTGAGAATTTGCCTC	-	101
orf327	GGGGATCAGTACAAGACGGC	GCAAGCATTAAGCCGTCGAA	-	114
orf345	GCTTTTGCCCATGCCATGAA	TCCCGGTAATTCCTTCCCCT	-	135
orf558	CCCGAATCGCTCGGTAAGAG	TTTCACTGCTACGCCAGCTT	-	134
5′ *nad6/orf891*	ACTTCTTCGTGAATCTCCGCT	TTCCCATCCCAGTCTTTCGC	163	163
3′ *nad6*	CAAGCCTGGACCCGCTATAC	TGGGAGGTCGGGGTATTCAA	94	-
3′ *orf891*	CAAGCCTGGACCCGCTATAC	TGGAGAGTTTCCGTGTAGAAGA	-	-*
orf933	AGCACTTTGGGCATTCCGT	ACAAGGGTTGGACCATCAGG	-	126
orf1197	AGTGGGCTAGGTACCACCAT	AAGCTTGACTCTATGCGCCA	-	140
atp1	CCCATGGCACAGCCAGAATA	CAGAAACGCTCAACTGTGGC	140	140

*151 bp amplicon was expected.

Suppl Table 2. The protein coding genes annotated in HA89(ANN2) mitochondrial genome.

Gene Name	Gene Features	Position in HA89(ANN2) Mitogenome	Orientation
	Complex I		
nad1	Encoding NADH-ubiquinone oxidoreductase chain 1. Trans-spliced gene, counting 4 exons	exon1 277278..277664	Plus

		exon2 41187..41267	Minus
		exon3 40142..40399	Minus
		exon4 109446..109697	Minus
nad2	Encoding NADH-ubiquinone oxidoreductase chain 2. Trans-spliced gene, counting 4 exons	exon 1 130673..130876	Plus
		exon 2 132080..132430	Plus
		exon 3 4997..5158	Minus
		exon 4 2049..2693	Minus
nad3	Encoding NADH-ubiquinone oxidoreductase chain 3	114556..114912	Plus
nad4	Encoding NADH-ubiquinone oxidoreductase chain 4. Gene consists of 2 exons	exon 1 226998..228021	Minus
		exon 2 229483..229943	Minus
nad4L	Encoding NADH-ubiquinone oxidoreductase chain 4L	28690..28962	Minus
nad5	Encoding NADH-ubiquinone oxidoreductase chain 5. Trans-spliced gene, counting 4 exons	exon1 104046..104243	Minus
		exon 2 105116..105511	Minus
		exon 3 293119..294333	Minus
		exon4 295182..295409	Minus
nad7	Encoding NADH-ubiquinone oxidoreductase chain 7. Gene consists of 4 exons	exon1 198509..198677	Plus
		exon2 201101..201542	Plus
		exon3 202607..202853	Plus
		exon4 204591..204905	Plus
nad9	Encoding NADH-ubiquinone oxidoreductase chain 9	120750..121322	Plus
	Complex III		
cob	Encoding cytochrome *b*	172018..173211	Minus
	Complex IV		
cox1	Encoding cytochrome *c* oxidase subunit 1	287502..289196	Plus
cox2	Encoding cytochrome *c* oxidase subunit 2. Gene consists of 2 exons	exon1 156440..156873	Plus
		exon2 158160..158604	Plus
cox3	Encoding cytochrome *c* oxidase subunit 3	218657..219454	Plus
	Complex V		
atp1	Encoding ATP synthase subunit 1	215911..217443	Plus
atp4	Encoding ATP synthase subunit 4	27935..28510	Minus
atp8	Encoding ATP synthase subunit 8	217608..218087	Plus
atp9	Encoding ATP synthase subunit 9	76854..77153	Plus

	Cytochrome c biogenesis		
ccmB	Encoding cytochrome *c* biogenesis B	247143..247763	Plus
ccmC	Encoding cytochrome *c* biogenesis C	15296..16039	Plus
ccmFC	Encoding cytochrome *c* biogenesis FC. Gene consists of 2 exons	exon1 168065..168833<br>exon2 169479..170329	Minus<br>Minus
ccmFN	Encoding cytochrome *c* biogenesis FN	205193..206911	Plus
	Ribosomal protein		
rpl5	Encoding ribosomal protein L5	223773..224336	Minus
rpl10	Encoding ribosomal protein L10	248071..248559	Minus
rpl16	Encoding ribosomal protein L16	90552..91067	Plus
rps3	Encoding ribosomal protein S3	88985..90661	Plus
rps4	Encoding ribosomal protein S4	68382..69377	Minus
rps11	Encoding ribosomal protein S11	289608..290027	Plus
rps12	Encoding ribosomal protein S12	114964..115341	Plus
rps13	Encoding ribosomal protein S13	42094..42444	Minus
	Gene with other functions and conservative ORFs with unknown function		
mttB	Encoding protein translocase component TatC. Gene consists of 2 exons	exon1 182524..183276<br>exon2 183749..183784	Minus<br>Minus
matR	Encoding intron maturase	110473..112440	Minus
orf873	Conservative ORF with unknown function. *According to genomic data has premature codon resulted in shorter transcript (orf618). However RNA editing may be involved, resulting in normal transcript	170474..171094*	Minus
	Newly detected ORFs		
orf324	ORF with unknown function	77915..78241	Minus
orf327	ORF with unknown function	84497..84826	Minus
orf345	ORF with unknown function	149669..150016	Minus
orf558	*cox2*-chimeric gene, encoding protein with transmembrane domain	295955..296515	Minus
orf891	*nad6*-chimeric gene, encoding protein with transmembrane domains	192053..192946	Plus
orf933	ORF with unknown function, encoding protein with transmembrane domains	82591..83526	Minus
orf1197	atp6-chemeric gene, encoding protein with transmembrane domains	146967..148166	Minus
